# GOLPH3 overexpression correlates with tumor progression and poor prognosis in patients with clinically N0 oral tongue cancer

**DOI:** 10.1186/1479-5876-10-168

**Published:** 2012-08-20

**Authors:** Huan Li, Ling Guo, Shu-Wei Chen, Xiao-Hui Zhao, Shi-Min Zhuang, Li-Ping Wang, Li-Bing Song, Ming Song

**Affiliations:** 1 State Key Laboratory of Oncology in South China, Guangzhou 510060, P.R. China; 2 Department of Head and Neck Surgery, Sun Yat-sen University Cancer Center, Guangzhou 510060, P.R. China; 3 Department of Nasopharyngeal Cancer, Sun Yat-sen University Cancer Center, Guangzhou 510060, P.R. China; 4 Department of Experimental Research, Sun Yat-sen University Cancer Center, Guangzhou 510060, P.R. China; 5 Department of Otolaryngology-Head & Neck Surgery, The Third Affiliated Hospital of Sun Yat-sen University, Guangzhou 510630, P.R. China

**Keywords:** GOLPH3, Prognosis, cN0 oral tongue cancer

## Abstract

**Background:**

Overexpression of GOLPH3 (Golgi phosphoprotein 3, 34 kDa) is associated with the progression of many solid tumor types leading to an unfavorable clinical outcome. We aimed to investigate the clinical significance of GOLPH3 expression in the development and progression of clinically N0 (cN0) oral tongue cancer.

**Methods:**

Real-time PCR and Western blotting analyses were employed to examine GOLPH3 expression in four oral tongue cancer cell lines, primary cultured normal tongue epithelial cells (TEC), eight matched pairs of oral tongue cancer samples and adjacent noncancerous tissue samples from the same patient. Immunohistochemistry (IHC) was performed to examine GOLPH3 protein expression in paraffin-embedded tissues from 179 cN0 oral tongue cancer patients. Statistical analyses were applied to evaluate the diagnostic value and the associations of GOLPH3 expression with clinical parameters.

**Results:**

GOLPH3 mRNA and protein was up-regulated in oral tongue cancer cell lines and cancerous tissues compared with that in primary cultured normal tongue epithelial cells (TEC) and adjacent noncancerous tissue samples. GOLPH3 protein level was positively correlated with clinical stage (*P* = 0.001), T classification (*P* = 0.001), N classification (*P* = 0.043) and recurrence (*P* = 0.009). Patients with higher GOLPH3 expression had shorter overall survival time, whereas those with lower GOLPH3 expression had longer survival time.

**Conclusion:**

Our results suggest GOLPH3 overexpression is associated with poor prognosis for cN0 oral tongue cancer patients and may represent a novel and useful prognostic indicator for cN0 oral tongue cancer.

## Background

Oral cancer is the tenth most commonly diagnosed cancer in men worldwide, and cancers of the oral cavity accounted for 263,900 cases worldwide in 2008 [1]. The tongue is the most cancer-prone intraoral site in most populations studied, and the most common pathological type of oral tongue cancer is squamous cell carcinoma [2]. Although intensive efforts have been made in primary prevention and improving therapy, morbidity and mortality rates for oral tongue cancer remain steadily high and are rising in a number of countries. Due to its highly invasive nature, oral tongue cancer frequently leads to severe defects in speech, mastication and deglutition, as well as cancer-related death. During the last three decades, the long-term survival rate for patients with oral tongue cancer has not improved substantially, and the tongue remains among the worst sites for all cancers [3]. In clinical practice, although head and neck surgeons mostly depend on the TNM classification system for planning treatment strategy, no consensus exists on the optimal treatment of the neck in cN0 oral tongue cancer patients [4]. However, the TNM system is not sufficiently reliable for predicting clinical outcome or for providing detailed information on the biological characteristics of a malignancy [5]. Recently, gene expression profiling has been reported to predict the clinical outcome more accurately than traditional clinical and pathological standards [6,7]. Therefore, the identification of genes associated with aggressiveness in cN0 oral tongue cancers is of great value in identifying high-risk patients who may benefit from more aggressive primary surgery or adjuvant treatment following surgery. Furthermore, this approach may also provide new targets for clinical intervention.

It is currently accepted that vesicular trafficking plays an important role in cancer development. Recently, this area of cancer research has focused on endocytic pathways regulating signal transduction cascades downstream of cell surface growth factor receptors [8]. These pathways play important roles in maintaining a balance of growth factor signaling and deregulated receptor trafficking, which could provide a mechanism to promote oncogenesis. The endocytic protein, GOLPH3, is a highly conserved 34 kDa protein initially identified through proteomic characterization of the Golgi apparatus. GOLPH3 binds to PtdIns(4)P-rich trans-Golgi membranes and MYO18A, conveying a tensile force required for efficient tubule and vesicle formation [9-11]. GOLPH3 gene expression was found to correlate with 5p13 copy number status in human lung cancer specimens, and functional studies (RNAi knockdown and cDNA overexpression) have shown that GOLPH3 is activated in cancers with 5p amplification and that it is a bona fide oncogene with potent transforming activity [12]. Importantly, the correlation between 5p13 copy number and increased phosphorylation of the p70 S6 kinase mTOR substrate in NSCLC tumor specimens links GOLPH3 function to mTOR activation [11] and indicates that GOLPH3 tumorigenesis may be mediated by mTOR signaling [13]. Although the mechanistic basis for GOLPH3 activation of mTOR signaling remains unclear, several lines of evidence suggest that GOLPH3 plays a role in vesicular trafficking and glycosylation, which are associated with oncogenicity. Thus, the regulation of mTOR by GOLPH3 may contribute to malignancy through these cellular pathways. GOLPH3 expression levels or copy number status may therefore serve as a useful prognostic factor for cancer.

In this study, we investigated GOLPH3 expression in a cohort of cN0 oral tongue cancers to determine the clinical significance of GOLPH3 overexpression in the development and progression of cN0 oral tongue cancer.

## Methods

### Patients and tissue specimens

The specimens were used with prior patients’ written consent and the approval of the Institutional Research Ethics Committee of the Sun Yat-sen University Cancer Center. A total of 187 tissue specimens were taken from patients with cN0 oral tongue cancer, none of whom had received radiotherapy or chemotherapy prior to surgery. For RT-PCR and Western blotting analysis, eight matched pairs of tumors tissue and adjacent noncancerous tissue samples were obtained from glossectomy specimens of patients diagnosed with cN0 oral tongue cancer immediately after surgery and stored at −80°C. The percentage tumor purity of these tissues was established by the histopathological analysis of adjacent sections prior to RNA and protein analysis. A total of 179 individual paraffin-embedded cN0 oral tongue cancer samples were obtained from 107 male and 72 female patients with a median age of 53 years (range 20 – 87 years), who had been diagnosed using clinical and histopathological methods at Departments of Head and Neck Surgery and Departments of Pathology, Sun Yat-sen University Cancer Center between 1998 and 2005. Clinical follow-up data was available for a minimum of 5 years or until death. Clinicopathological and immunohistochemical analyses of these samples were performed to determine the prognostic significance of GOLPH3 expression. All patients received standard therapy based on the clinical stage. In brief, patients with early-stage tumors (stages I and II) received surgery alone, whereas those with advanced-stage cancer (stages III and IV) received combination therapy comprising surgery and radiotherapy. Patient progress was followed for 78.3 ± 42.1 months (mean ± SD). The clinical information this patient cohort is summarized in Table1.

**Table 1:** Clinicopathologic characteristics and GOLPH3 expression of patients with clinically N0 oral tongue cancer of the study cohort (n = 179)

**Characteristics**	**Number of cases (%)**
**Gender**	
Male	109 (60.9)
Female	70 (39.1)
**Age (years)**	
< 53	89 (49.7)
≥ 53	90 (50.3)
**Pathologic stage**	
**I**	80 (44.7)
**II**	67 (37.4)
**III**	17 (9.5)
**IV**	15 (8.4)
**T classification**	
T1	87 (48.6)
T2	87 (48.6)
T3	5 (2.8)
**N classification**	
N0	149 (83.2)
N1	15 (8.4)
N2	15 (8.4)
**Nodal status**	
N0	149 (83.2)
N + (N1 and N2)	30 (16.8)
**Pathologic differentiation**	
Well	136 (76.0)
Moderately	36 (20.1)
Poorly	7 (3.9)
**Recurrence**	
No	123 (68.7)
Yes	56 (31.3)
**Vital status (at follow-up)**	
Alive	131 (73.2)
Dead	48 (26.8)
**Expression of GOLPH3**	
Low or none expression	57 (31.8)
High expression	122 (68.2)

### Cell lines

Four human oral tongue cancer cell lines were purchased from the American Type Culture Collection (SCC-25 and CAL-27) and the Cell Bank of Type Culture Collection of Chinese Academy of Sciences (TSCCa and Tca-8113). SCC-25 and CAL-27 cells were grown in DMEM (Invitrogen) supplemented with 10% fetal bovine serum (HyClone Laboratories, Logan, UT). TSCCa and Tca8113 cells were cultured in RPMI-1640 medium (GIBCO BRL, Rockville, MD) supplemented with 10% fetal bovine serum (HyClone Laboratories, Logan, UT). Primary cultured normal tongue epithelial cells (TEC) were established from tissue obtained during a glossectomy for a benign lesion and maintained in Keratinocyte-SFM (Gibco, Invitrogen Corp, USA).

All cells were grown in 5% CO_2_ in a humidified atmosphere at 37°C.

### Real-time PCR (RT-PCR)

Total RNA was extracted from cultured cells and fresh tissues using TRIzol reagent (Invitrogen) according to the manufacturer’s instruction and treated with RQ1 RNase-free DNase (Promega). cDNA was synthesized from 2 μg RNA using a iScript™ cDNA Synthesis Kit (Bio-Rad Laboratories) and the quantitation of GOLPH3 mRNA was performed by qPCR using a SsoFast EvaGreen Supermix (Bio-Rad Laboratories and a Bio-Rad CFX96 sequence detection system). Cycling condition included initial denaturation at 95°C for 30s followed by 40 cycles of 95°C for 5 s and 60°C for 5 s. Primers for GOLPH3 and β-actin were designed using Primer Express Software v. 2.0 (Applied Biosystems). GOLPH3 expression data was normalized to β-actin and all experiments were performed in triplicate.

### Western blotting

Cells were washed twice with ice-cold phosphate-buffered saline (PBS) and lysed on ice in RIPA (radio immunoprecipitation assay) buffer (Cell Signaling Technology, Danvers, MA) containing complete protease inhibitor cocktail (Roche Applied Science, Mannheim, Germany). Fresh tissue samples were ground to powder in liquid nitrogen and lysed with SDS-PAGE sample buffer. Protein samples (20 μg) were separated on 12% SDS-polyacrylamide gels and transferred to PVDF membranes (Immobilon P, Millipore, Bedford, MA).

Membranes were blocked with 5% fat-free milk in Tris-buffered saline containing 0.1% Tween-20 (TBST) for 1 h at room temperature. Membranes were incubated with anti-GOLPH3 antibody (1:1000, Abcam, ab69179) overnight at 4°C, then with horseradish peroxidase-conjugated goat anti-rabbit IgG (Santa Cruz Biotechnology, SC-2004), and GOLPH3 expression was detected using ECL prime Western blotting detection reagent (Amersham) according to the manufacturer’s instructions. β-actin was used as a loading control.

### Immunohistochemistry

Immunohistochemical analysis was used to measure GOLPH3 protein expression in 179 human oral tongue cancer tissues. Immunohistochemistry methods and scoring for GOLPH3 expression were done as previously described. Briefly, paraffin-embedded specimens were cut into 4 μm sections and baked at 60°C for 2 h followed by deparaffinization with xylene and rehydration. Sections were then submerged in EDTA antigenic retrieval buffer and microwaved for antigenic retrieval, treated with 3% hydrogen peroxide in methanol to quench endogenous peroxidase activity, and incubated with 1% bovine serum albumin to block nonspecific binding. Sections were then incubated with anti-GOLPH3 rabbit polyclonal antibody (1:100, Abcam, ab69179) at 37°C for 40 min. Normal goat serum was used as a negative control. After washing, tissue sections were incubated with biotinylated anti-rabbit secondary antibody (Zymed), then with streptavidin-horseradish peroxidase complex (Zymed). Finally, sections were immersed in 3.3′-diaminobenzidine, counterstained with 10% Mayer’s hematoxylin, dehydrated, and mounted.

GOLPH3 staining was scored independently by two pathologists. The proportion of positive tumor cell was scored as: 0, no positive tumor cells; 1, 1%–10% positive tumor cells; 2, 11%–35% positive tumor cells; 3, 36%–70% positive tumor cells; and 4, >70% positive tumor cells. Staining intensity was scored as: 0, no staining; 1, weak staining (light yellow); 2, moderate staining (yellow brown); and 3, strong staining (brown). The staining index for GOLPH3 expression in oral oral tongue cancer lesions was calculated by multiplying the two scores obtained for each sample and obtained values of 0, 1, 2, 3, 4, 6, 9, or 12. A score of >6 was defined as high GOLPH3 expression and scores of <4 defined low GOLPH3 expression.

### Statistical analyses

All statistical analyses were carried out using the SPSS software package (IBM, standard version 17.0). Pearson’s χ^2^ and Fisher’s exact tests were used to analyze the relationship between GOLPH3 expression and clinicopathological characteristics. Overall survival was defined as the time from surgery to death, and disease-free survival was defined as the time from surgery to the onset of recurrence (diagnosed by clinical assessment or imaging). Kaplan–Meier survival curves were plotted and compared using a log-rank test. Multivariate survival analysis was performed for all parameters found to be significant in the univariate analysis using the Cox regression model. A two-sided probability value of less than 0.05 was considered to be statistically significant.

## Results

### GOLPH3 is overexpressed in oral tongue cancer cell lines

**Figure 1:**
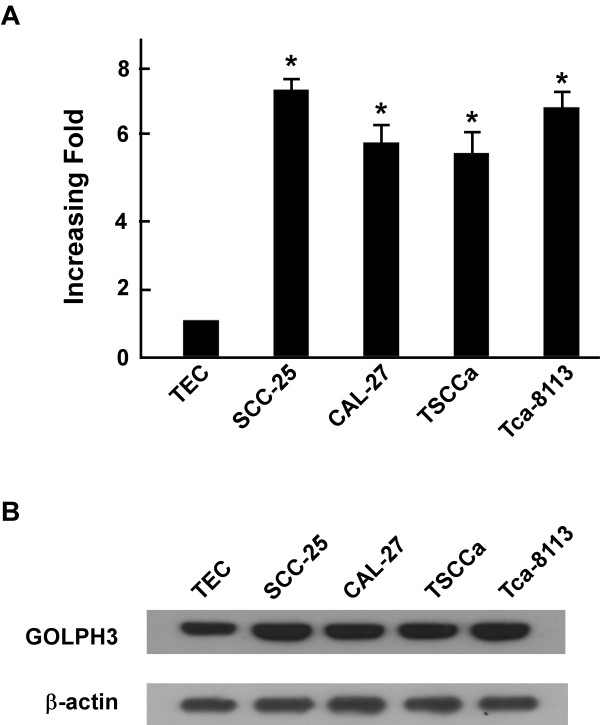
**Overexpression of GOLPH3 mRNA and protein in tongue cancer cell lines.** (**A** and **B**) Expression of GOLPH3 mRNA and protein in tongue cancer cell lines (SCC-9, SCC-25, CAL-27, TSCCa, Tca-8113) and TEC were examined by qPCR (**A**) and Western blotting (**B**). Expression levels were normalized to β-actin. Error bars represent standard deviation of the mean (SD) calculated from three parallel experiments.

In order to investigate the potential role of GOLPH3 in the tumorigenesis of oral tongue cancer, the expression of GOLPH3 mRNA and protein were determined for four oral tongue cancer cell lines (SCC-25, CAL-27, TSCCa and Tca-8113) and compared with GOLPH3 expression in primary cultured normal tongue epithelial cells (TEC). GOLPH3 mRNA expression was at least 5-fold higher in oral tongue cancer cell lines than that in TEC (Figure 1A), and GOLPH3 protein was highly expressed in oral tongue cancer cell lines and only weakly expressed in TEC (Figure1B).

### GOLPH3 is overexpressed in oral tongue cancer tissues

**Figure 2:**
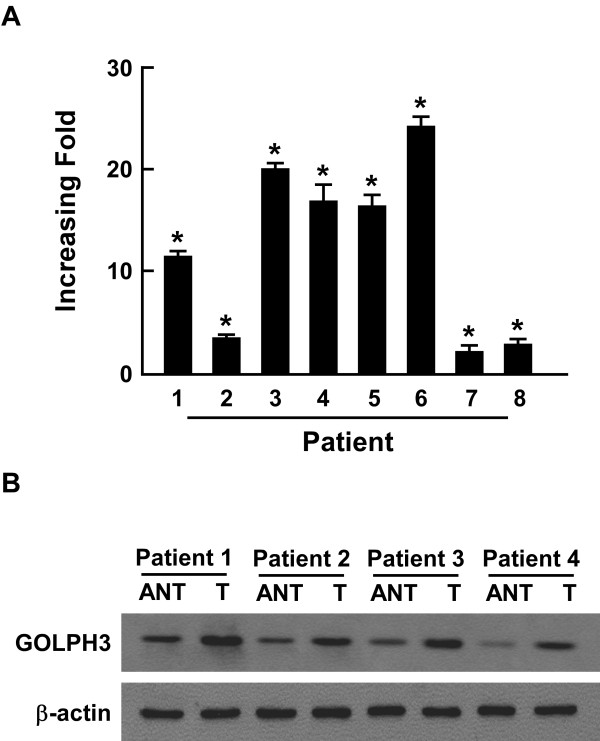
**The overexpression of GOLPH3 protein was in tongue cancers.** (**A**) Average T/N ratios of GOLPH3 mRNA expression in paired tongue cancer (T) and adjacent noncancerous tissues (N) was quantified by qPCR and normalized to β-actin. Error bars represent standard deviation of the mean (SD) calculated from three parallel experiments. (**B**) Representative Western blotting analyses images of GOLPH3 protein expression in four matched pairs of oral tongue cancer (T) and adjacent noncancerous tissues (N). β-actin was the loading control.

To investigate GOLPH3 mRNA and protein expression in cN0 oral tongue cancer, RT-PCR and Western blotting analyses were done on eight matched pairs of oral tongue cancer samples (T) and adjacent noncancerous tissue samples (N). GOLPH3 mRNA was expressed at higher levels in all oral tongue cancer tissue samples than that in adjacent noncancerous tissues, with differential expression ranging from 2.2-fold to 24.1-fold (Figure 2A). Consistent with this data, GOLPH3 protein was also up-regulated in cN0 oral tongue cancers compared with the matched controls (Figure2B).

### GOLPH3 overexpression is associated with clinical features of cN0 oral tongue cancer

**Figure 3:**
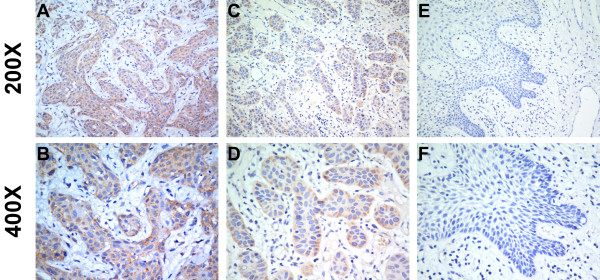
**The expression of GOLPH3 protein in oral tongue cancer sections.** Representative immunohistochemical images of cN0 oral tongue cancer tissue specimens indicating strong GOLPH3 staining (**A** and **B**); moderate GOLPH3 staining (**C** and **D**); and weak or negative detectable GOLPH3 staining (**E** and **F**). Magnification is × 200 (**A**, **C** and **E**) or × 400 (**B**, **D** and **F**).

We further investigated the link between GOLPH3 protein expression and the clinicopathological characteristics of oral tongue cancer using a panel of 179 paraffin-embedded, archived oral tongue cancer specimens, including 80 stage I tumors, 67 stage II tumors, 17 stage III tumors, and 15 stage IV tumors. GOLPH3 expression was analyzed by immunohistochemical staining with an anti-GOLPH3 antibody. As shown in Table1, 167 of the total 179 oral tongue cancers (93.2%) were positive for GOLPH3 based on immunohistochemical staining. High GOLPH3 protein expression was detected in 122 samples (68.2%) and weak or negative staining was observed in 57 tumor samples (31.8%, Figure 3).

Statistical analysis showed a strong correlation between GOLPH3 expression, as determined using immunohistochemical staining, and clinicopathological characteristics of cN0 oral tongue cancer, including clinical stage (*P* =0.001), T classification (*P* = 0.001), N classification (*P* =0.043), and recurrence (*P* =0.021). In contrast, GOLPH3 expression did not correlate with age, gender and tumor differentiation (Table2). Furthermore, Spearman correlation analysis determined that the level of GOLPH3 overexpression correlated with clinical stage (*P* < 0.001), T classification (*P* < 0.001), N classification (*P* = 0.021), recurrence (*P* = 0.001) and vital status (*P* = 0.01). Taken as a whole, our data shows that GOLPH3 protein overexpression positively correlates with pathological stage, T classification, N classification, recurrence and vital status, and GOLPH3 overexpression occurs during the clinical progression of oral tongue cancer.

**Table 2:** Correlation between GOLPH3 expression and clinicopathologic characteristics of patients with clinically N0 oral tongue cancer

**Characteristics**	***n***	**GOLPH3 expression**		**χ**^**2**^**test*P***
		**Low or none, no. (%)**	**High, no. (%)**	**(Fisher’s exact test*P*)**
**Gender**				0.574
Male	109	33 (30.3)	76 (69.7)	
Female	70	24 (34.3)	46 (65.7)	
**Age (years)**				0.594
< 53	89	30 (33.7)	59 (66.3)	
≥ 53	90	27 (30.0)	63 (70.0)	
**clinical stage**				**0.001**
I	80	39 (48.8)	41 (51.2)	
II	67	14 (20.9)	53 (79.1)	
III	17	1 (5.9)	16 (94.1)	
IV	15	3 (20)	12 (80)	
**T classification**				**0.001**
T1	87	39 (44.8)	48 (55.2)	
T2	87	16 (18.4)	71 (81.6)	
T3	5	2 (40.0)	3 (60.0)	
**N classification**				**0.043**
N0	149	53 (35.6)	96 (64.4)	
N1	15	1 (6.7)	14 (93.3)	
N2	15	3 (20.0)	12 (80.0)	
**Pathologic differentiation**				**0.338**
Well	136	46 (33.3)	90 (66.7)	
Moderately	36	8 (22.2)	28 (77.8)	
Poorly	7	3 (42.9)	4 (57.1)	
**Recurrence**				**0.009**
No	120	45 (37.5)	75 (62.5)	
Yes	59	11 (18.6)	48 (81.4)	
**Vital status (at follow-up)**				**0.010**
Alive	122	46 (37.8)	76 (62.2)	
Dead	57	11 (19.3)	46 (80.7)	

### Association between GOLPH3 expression and patient survival

**Figure 4:**
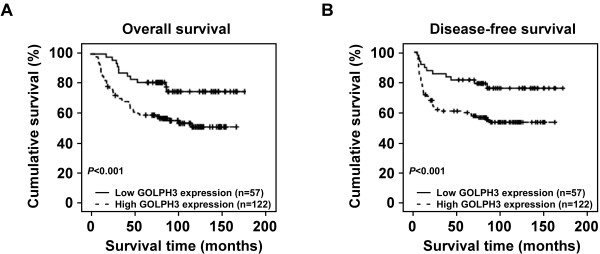
**The level of GOLPH3 protein expression affects overall survival and disease-free survival.** Kaplan–Meier curves with univariate analysis (log-rank) for cN0 oral tongue cancer patients with high GOLPH3 expression (n = 57) versus low or no GOLPH3 expression (n = 122) for overall survival (**A**) and disease-free survival (**B**).

Patient survival analysis showed a clear negative correlation between the level of GOLPH3 protein expression and both the overall survival and disease-free survival of patients with cN0 oral tongue cancer (*P* = 0.010 and 0.009, respectively; Figure 4A, B). The cumulative 5-year overall and disease-free survival rates for patients with high levels of GOLPH3 expression were found to be 62.3% and 61.5%, respectively, whereas for patients with low or no GOLPH3 expression the rates were 80.7% and 80.4%, respectively. Cox regression revealed that only N classification (relative risk, 1.859, CI: 1.353–2.559, *P* < 0.001) and GOLPH3 overexpression (relative risk, 2.064, CI: 1.061–4.015, *P* = 0.033) were independent prognostic factors for poor overall survival.

## Discussion

As the most common cancer diagnosed in the oral cavity, tongue squamous cell carcinomas comprise 25%–40% of all oral carcinomas [14]. The poor prognosis of oral tongue cancer is mainly a consequence of its unusual histological makeup (including a rich lymphatic network and a highly muscularized structure), which makes it poorly equipped to resist invasion and metastasis [15]. In clinical practice, the most important prognostic factors are tumor size, nodal involvement, and depth of infiltration, although this system cannot reliably predict the clinical outcome or provide useful information concerning the biologic characteristics of the malignancy [5]. Although some biomarkers correlate with the prognosis of oral tongue cancer, no reliable prognostic biomarkers for oral tongue cancer are available for clinical use. Improving prognostic markers are urgently needed, as survival rates for patients with tumors at the same clinicopathological stage vary considerably.

In the current study, we have demonstrated the clinical significance of GOLPH3 overexpression in cN0 oral tongue cancer for the first time. We also investigated the potential for GOLPH3 expression level to be a clinical prognostic indicator for disease progression and patient survival in cN0 oral tongue cancer. We found that GOLPH3 was highly expressed in cN0 oral tongue cancer cell lines and tissue samples at both the transcriptional and translational levels, and that GOLPH3 protein overexpression correlated with the clinical features of cN0 oral cancer, including clinical stage, T classification, N classification, nodal status, vital status and prognosis. Furthermore, the cumulative 5-year overall and disease-free survival rates of patients with high GOLPH3 expression are lower than those with low or undetectable GOLPH3 expression. Thus, patients with high GOLPH3 expression have a poorer prognosis than those with low or absent GOLPH3 expression, making GOLPH3 a potential independent prognostic factor for cN0 oral tongue cancer.

GOLPH3 was originally identified following proteomic characterization of the Golgi apparatus, and GOLPH3 protein binds to PtdIns(4)P-rich trans-Golgi membranes and MYO18A to provide a tensile force required for efficient tubule and vesicle formation [9-11]. GOLPH3 plays an important role in malignant transformation and cell growth by regulating the localization of protein glycosyltransferases to the Golgi [16,17]. Recent studies identified a role for GOLPH3 in regulating various biological processes during tumorigenesis and GOLPH3 has been associated with the progression and outcome of many tumor types. An increasing number of studies have found GOLPH3 upregulation in several types of cancers, thus indicating a role for GOLPH3 as a positive regulator of cancer progression.

Furthermore, GOLPH3 overexpression correlates with hyperactivation of mTORC2 and mTORC1 signaling in human cells [12]. Xenograft experiments revealed that tumor cells overexpressing GOLPH3 have an increased sensitivity to the mTORC1 inhibitor, rapamycin, and GOLPH3-dependent oncogenesis is associated with increased mTOR signaling [13]. The serine/ threonine protein kinase, mTOR, is a primary regulator of protein synthesis and cell growth that integrates diverse upstream signals including amino acid and energy stress sensing to regulate cell proliferation, growth and survival. The regulation of cell size by mTOR[18-20] may be important for cancer development, progression, and metastasis. Cell growth, proliferation, and survival are regulated by a complex network of intracellular and extracellular signal transduction cascades. The growth factor-responsive receptor tyrosine kinase (RTK) phosphatidylinositol 3-kinase (PI3K) pathway plays a key role in governing these processes [15]. In addition, the serine/threonine kinase AKT functions as a central integrator of RTK–PI3K signaling to modulate downstream effectors, notably the TSC1/2-mTOR complexes. GOLPH3 can enhance downstream growth signaling in response to RTK activation [13]. We therefore hypothesize that GOLPH3 may affect the development and progression of cN0 oral tongue cancer through the PI3K–AKT–mTOR signaling pathway.

In this study, we investigated GOLPH3 mRNA and protein expression levels in a series of cN0 oral tongue cancer samples. We found that GOLPH3 was highly expressed in cN0 oral tongue cancer cell lines and tissues at both the transcriptional and translational levels, consistent with the hypothesis that GOLPH3 is an oncogene. Elevated levels of GOLPH3 protein positively correlated with several clinicopathologic characteristics of cN0 oral tongue cancer, including pathological stage, T classification, N classification, and nodal status. Moreover, cN0 oral tongue cancer patients with increased GOLPH3 expression had significantly shorter overall and disease-free survival time than patients with lower or no GOLPH3 expression (*P* = 0.010 and *P* = 0.009, respectively). We therefore report that GOLPH3 is a risk factor for cN0 oral tongue cancer, as the upregulation of GOLPH3 in cN0 oral tongue cancer patients indicates a poor prognosis. Thus, the detection of overexpressed GOLPH3 in cN0 oral tongue cancer should identify high-risk tumor phenotypes that require more aggressive primary surgery or adjuvant treatment following surgery. However, while our studies offer some insight into the function of GOLPH3 in tongue squamous cell carcinoma, the underlying mechanism of GOLPH3-mediated oral tongue cancer progression, the role of GOLPH3 in malignant transformation and cell growth and its effects on clinical outcome remain to be defined.

In conclusion, we have demonstrated an important role for GOLPH3 in cN0 tongue carcinogenesis. We suggest that determining GOLPH3 expression levels in cN0 oral tongue cancers may help to identify patients harboring occult micrometastases that require more aggressive treatment and may therefore complement the current TNM classification to enable better risk stratification and election for adjuvant therapy. We further propose that targeting GOLPH3 may be a useful strategy for developing novel therapeutic modalities.

## Conclusions

In this study, we found that up-regulation of GOLPH3 correlated with poor prognosis and reduced survival of patients with cN0 oral tongue cancer. Multivariate analysis showed that GOLPH3 protein levels could be used as an independent prognostic predictor for cN0 oral tongue cancer patients. Thus, testing the GOLPH3 protein level may be useful for formulating prognosis and guiding the follow-up schedule in patients with cN0 oral tongue cancer.

## Competing interests

The authors declare that they have no competing interests.

## Authors’ contributions

HL carried out Immunohistochemical (IHC) analysis, drafted the manuscript. LG and SC collected the tissue specimens and patient information, and carried out the statistical analyses. XZ carried out the Western blotting, RNA extraction and real-time PCR. SZ and LW participated in collecting patient information and editing of the manuscript. LS participated in conceiving the study and guiding the editing of the manuscript. MS conceived the study, wrote and guided the editing of the manuscript. All authors read and approved the final manuscript.
